# Ankle Injury Management (AIM): design of a pragmatic multi-centre equivalence randomised controlled trial comparing Close Contact Casting (CCC) to Open surgical Reduction and Internal Fixation (ORIF) in the treatment of unstable ankle fractures in patients over 60 years

**DOI:** 10.1186/1471-2474-15-79

**Published:** 2014-03-12

**Authors:** Keith Willett, David J Keene, Lesley Morgan, Bridget Gray, Robert Handley, Tim Chesser, Ian Pallister, Elizabeth Tutton, Christopher Knox, Ranjit Lall, Andrew Briggs, Sarah E Lamb

**Affiliations:** 1 Nuffield Department of Orthopaedics, Rheumatology and Musculoskeletal Sciences, Kadoorie Centre for Critical Care Research and Education, John Radcliffe Hospital, University of Oxford, Oxford OX3 9DU, UK; 2 Oxford Trauma Service, John Radcliffe Hospital, Oxford University Hospitals NHS Trust, Oxford OX3 9DU, UK; 3 Department of Orthopaedic Surgery, Frenchay Hospital, North Bristol NHS Trust, Bristol BS16 1LE, UK; 4 Department of Orthopaedic Surgery, Morriston Hospital, Swansea SA6 6NL, UK; 5 Warwick Clinical Trials Unit, Warwick Medical School, University of Warwick, Coventry CV4 7AL, UK; 6 Faculty of Medicine, University of Glasgow, Glasgow G12 8RZ, UK

**Keywords:** Ankle, Fracture, Trauma, Orthopaedics, Cast, Surgery, Conservative, Operative, Adult

## Abstract

**Background:**

Ankle fractures account for 9% of all fractures with a quarter of these occurring in adults over 60 years. The short term disability and long-term consequences of this injury can be considerable. Current opinion favours open reduction and internal fixation (ORIF) over non-operative treatment (fracture manipulation and the application of a standard moulded cast) for older people. Both techniques are associated with complications but the limited published research indicates higher complication rates of fracture malunion (poor position at healing) with casting. The aim of this study is to compare ORIF with a modification of existing casting techniques, Close Contact Casting (CCC). We propose that CCC may offer an equivalent functional outcome to ORIF and avoid the risks associated with surgery.

**Methods/Design:**

This study is a pragmatic multi-centre equivalence randomised controlled trial. 620 participants will be randomised to receive ORIF or CCC after sustaining an isolated displaced unstable ankle fracture. Participants will be recruited from a minimum of 20 National Health Service (NHS) acute hospitals throughout England and Wales. Participants will be aged over 60 years and be ambulatory prior to injury. Follow-up will be at six weeks and six months after randomisation. The primary outcome is the Olerud & Molander Ankle Score, a functional patient reported outcome measure, at 6 months. Follow-up will also include assessments of mobility, ankle range of movement, health related quality of life and complications. The six-month follow-up will be conducted face-to-face by an assessor blinded to the allocated intervention. A parallel economic evaluation will consider both a health service and a broader societal perspective including the individual and their family. In order to explore patient experience of their treatment and recovery, a purposive sample of 40 patients will also be interviewed using a semi-structured interview schedule between 6-10 weeks post treatment.

**Discussion:**

This multicentre study was open to recruitment July 2010 and recruitment is due to be completed in December 2013.

**Trial registration:**

Current Controlled Trials ISRCTN04180738.

## Background

In 2009/10 Hospital Episode Statistics for the National Health Service (NHS) in the United Kingdom recorded 343,536 fractures that required admission or surgery. Ankle fractures account for 9% of all fractures [1] and have an increasing incidence in the population aged over 50 years, with the trend set to continue [2,3]. A Scottish study reported an incidence of 132 fractures per 100,000 in men, and 112 fractures per 100,000 in women, per year, with the highest incidence of 248 per 100,000 per year occurring in women between the ages of 75 and 84 [3]. A three-fold increase is predicted from 2000 to 2030 as the population ages [4]. Ankle fractures have a substantial and lasting impact on mobility and related functions (e.g. standing, walking and stair climbing), with older adults often having the worse outcomes [5-9]. This can affect the ability to live independently.

For the young adult patient the established treatment is open reduction and internal fixation (ORIF), in which the bone fragments are repositioned at surgery and held in place until healing (union) by plates and/or screws. The incidence of wound problems in patients under 60 years of age undergoing surgery is also low (typically 1-5% of cases). It is more difficult to achieve successful surgical outcome in older people (adults aged over 60 years), because of a higher prevalence of co-morbidities resulting in lower bone density, frail skin and impaired wound healing. Poor bone quality (resulting from osteoporosis) directly affects the efficacy of stabilisation treatment methods for the bone fracture fragments [10]. Such fractures, because of the greater fragmentation and poor bone strength, tend to be less stable after repositioning and the holding strength of fixation screws can be diminished up to 10 fold [11]. This can render fixations incompetent and prevent early joint movement and weight-bearing – the accepted advantages of the surgical fixation approach in the younger patient. Other common co-morbidities in the older patient (peripheral vascular disease, chronic venous insufficiency, late onset diabetes, and/or oedema from heart failure) directly affect the lower limb skin and soft tissue tolerance of surgical wounds or traditional casts.

In older people, rates of postoperative complications from ankle fracture fixation surgery are high, for example 33% of patients required reoperation for removal of implants in one study [12]. Soft tissue complications after surgery also have a negative effect on long-term functional outcome [13]. Other case series support ORIF for a more predictable good outcome and acceptably low complication rates [14,15]. Despite the common occurrence of ankle fractures in older people published research is considered low quality [16]. Non-consecutive case series, non-randomised and retrospective studies dominate. Follow up is often incomplete, and there is a reliance on data extracted from records and radiographs as opposed to the patient-important and functional outcomes. A 2012 Cochrane review [17], comparing surgical versus non-operative treatment for ankle fractures, included 292 participants in three randomised controlled trials [18-20] and one quasi-randomised controlled trial [21]. The authors concluded that there is currently insufficient evidence of the effects of surgical versus conservative treatment on outcome after ankle fracture and confirmed the need for an adequately powered clinical trial.

Currently ORIF is more commonly used than non-operative treatment for ankle fracture in older people. Both are associated with complications but the limited published research indicates higher rates of loss of fracture reduction or malunion (poor position at healing) with traditional casting [17]. Traditional casting methods (an external support formed by an under layer of stockinette, layers of wool roll and felt, and a rigid outer layer made of plaster of Paris and/or synthetic material) can also create pressure sores. Many surgeons make a clinical judgement alone on a) the likely tolerance of a patient’s skin for surgical incisions and b) the bone quality and chance of achieving implant fixation. For patients judged as higher risk for open surgery, some surgeons may select manipulation and traditional casting, assuming fewer complications but with a higher risk of malunion.

A modification of the traditional casting treatment, with the potential of improved fracture stabilisation and lower skin damage risk, has now been identified – Close Contact Casting (CCC). This is a modification of “total contact casting” used extensively and successfully for more than 20 years in treating leg ulcers in diabetics who have frail skin [22-24]. CCC works by creating an intimate, anatomic, very close fit to the lower leg shape so dissipating forces evenly over all the skin, avoiding high local contact areas, protecting and promoting skin recovery.

A feasibility study has confirmed the viability of the study design and outcome measures proposed. It has also provided data to inform the estimates of sample size, along with recruitment rates. A parallel vascular laboratory investigation has confirmed the potential for improved skin viability outcomes with CCC [25]. There is a timely need for a properly constructed randomised controlled trial comparing CCC to ORIF, both for patient-important outcomes and cost effectiveness. This research question was a product of a research priority setting exercise undertaken with the orthopaedic trauma surgeon members of the UK Association for Osteosynthesis (AOUK) to identify research areas of importance for surgical fracture treatment [26].

### Primary objective

To determine if the application of the Close Contact Casting technique (CCC) for displaced ankle fractures in older adults results in an equivalent outcome compared to the standard care of open surgical reduction and internal fixation (ORIF) in terms of function, complications, quality of life and patient satisfaction with treatment.

### Secondary objectives

An economic evaluation will run in parallel to the study and will consider the costs of the two treatments to (i) the NHS, and (ii) the broader societal perspective including to the individual and their family. Potential complications, readmissions, revision surgery rates and mortality will be monitored carefully and considered in the overall appraisal of clinical and cost effectiveness.

## Methods/Design

### Summary of study design

A pragmatic multi-centre randomised controlled equivalence trial with parallel prospective economic evaluation. Participants will be randomised to receive ORIF or CCC after admission for surgery for displaced unstable ankle fractures in the Trauma and Orthopaedic Surgery departments of a minimum of 20 NHS acute hospitals.

### Study participants

Men or women aged over 60 years with displaced unstable fracture of the ankle who meet the eligibility criteria:

### Inclusion criteria

● Men or women aged over 60 years

● Isolated displaced unstable ankle fracture

● Ambulatory prior to the injury - in any capacity

● Capable of giving informed consent

● Capable of adhering to post-operative instructions

● Resident within the catchment area of a recruiting hospital

● Can attend for 6-month follow up

### Exclusion criteria

● Established critical limb ischaemia

● Insulin dependent diabetes mellitus

● Active leg ulceration

● Open fractures

● Serious concomitant disease - metastatic disease or terminal illness

● Clinically substantial degenerative or inflammatory arthritis (in the ankle)

● Unfit for general anaesthetic

● Cognitive impairment - Mini-Mental State Exam (MMSE) of under 16/30 [27]

● Patient unwilling to give informed consent.

### Participant approach and recruitment

The treating surgical team will undertake the initial approach to participants. It will be important at this stage that clinicians do not inadvertently influence potential participants by describing or emphasising only one of the possible treatment options. If the participant is willing, a member of the research team will explain the study in more detail and check eligibility criteria. A Mini-Mental State Examination (MMSE) to assess cognitive function will be undertaken prior to randomisation (see eligibility criteria). Potential participants will be given as long a time as possible to consider participation; traditionally treatment is delayed a few days to allow injury swelling to settle.

### Interventions

The surgical techniques, designated by the study, are common in UK surgical practice and lie within the expertise of UK trained orthopaedic surgeons. Training on Close Contact Casting (CCC) will be provided for surgeons by a member of the trial team. All cases will conform to the NHS standard of being performed under consultant supervision. Both study interventions are applied in theatre under general or regional anaesthesia. We will record time to treatment and type of anaesthesia (regional, general or both). Surgeons will be advised that talar tilt or shift resulting in significant joint incongruence would be considered unacceptable but as a pragmatic trial this will ultimately be a local clinical decision. Prior to definitive intervention, patients typically have their ankle immobilised in a type of temporary cast (or less frequently with an external fixator).

### Standard care

#### Open surgical reduction and internal fixation (ORIF)

Surgeons will be permitted to choose from the range of implants that are used in the UK, and will comply with internationally recognised AO principles of Fracture Management [28].

### Intervention

#### Manipulation under anaesthetic in theatre and application of Close Contact Cast (CCC)

Standardisation of the casting materials, cast design and application, and moulding technique will be achieved by surgeon instruction (training session and access to training videos and documentation). The method of closed fracture manipulative reduction of deformity will be at the discretion of individual surgeons and this falls within the common contemporary skills set of senior surgical trainees and consultants. The CCC is applied once major swelling has subsided at a similar time to that when open surgery would be considered. The use of specific moulding points and sited pressure pads prevents fracture displacement whilst minimising the risk of skin damage. In the weeks following the initial cast application, and after any re-applications of the cast (if required), participants in the CCC group will require monitoring X-rays to check maintenance of fracture position.

It is possible that in some cases, after randomisation, the intervention delivered will necessarily be changed. We anticipate the following:

● After randomisation at the point of intervention with anaesthesia commenced, the temporary cast is removed. The ankle skin condition may have deteriorated such that the surgeon considers one or all necessary surgical incisions to be unsafe. If randomised to ORIF, an alternative treatment* would be given.

● After randomisation at the point of intervention with anaesthesia commenced, a fracture may prove irreducible by closed manipulation. The surgeon would necessarily proceed to open surgical reduction. If that is required, internal fixation would be undertaken.

● If there is an unacceptable loss of position by either treatment method prior to fracture healing, the surgeon will adopt the treatment approach* best judged to achieve a favourable outcome.

● Very rarely a combination of bone and skin fragility and gross joint instability will exclude either intervention. The surgeon will apply a temporising external fixator and definitive treatment* will be at the surgeon’s discretion.

* alternative treatments include: i) traditional plaster cast, ii) external fixation iii) ORIF. CCC will be excluded as an option outside the group randomised to CCC.

### Standardisation of other treatments

Each hospital will follow its own antibiotic prophylaxis protocol for the type of implant insertion procedure for the ORIF group. No antibiotics will be routinely administered to the CCC patients in theatre. Thromboprophylaxis will reflect local hospital policy. In line with normal practice, the expectation is the majority of participants will not begin partial or full weight-bearing until at least 4 weeks after intervention [17,29]. However, the post-operative management plan, including the progression of weight-bearing, will be at the discretion of the individual surgeon. Rehabilitation will focus on early restoration of independent mobility, in as timely a fashion as possible. All participants will be reviewed by an orthopaedic surgeon independent to the study team to deal with any on-going symptoms such as pain. It will not be possible to blind the treating surgeon or radiograph assessors. The implants, or their absence, will be apparent on the radiographs as will the soft tissue scars on examination.

### Learning and expertise effects

This is a pragmatic study. We will monitor and analyse data to establish the extent, if any, of learning or expertise effects. It is common practice that surgeons have particular expertise in selected techniques, and for surgical teams to organise their workloads so that expertise is utilised to best effect. This study will not interfere with this dynamic. It is therefore not easy to anticipate the direction of expertise and learning effects. For each surgeon participating in the study, we will collect the following information: historical experience and preferences for ORIF and casting, grade of surgeon, time since first operation on the study and, if applicable, years since obtaining a certificate of completion of specialist training. These data will be summarised by recruitment centre and by treatment group and appropriate summary statistics will be produced including tests of association between surgeon experience and the procedures carried out. This will then guide recommendations on implementation and training if the technology proves effective.

### Baseline assessments

Baseline assessments will be undertaken by one of the research team following consent and prior to randomisation. None of the participants will be ambulatory at the baseline phase, but we will collect information about pre-injury mobility status using a retrospective report from the Olerud & Molander ankle score [30] as well as health related quality of life using the EQ-5D [31] and SF-12 [32]. Although not ideal, recall is the only method that we will have of assessing pre-fracture abilities. As the recall period is relatively short, typically a couple of days (up to two weeks), we do not anticipate problems. The type of residence in the month prior to admission will be recorded, as will the level of support provided and whether the participants lived alone prior to the injury. Information on pre-injury mobility status, medical history, smoking, alcohol intake, allergies, medication and care requirements will be collected.

### Outcome measures

Participants will be asked to attend study assessments at six weeks and six months. The outcomes and time points for the study are outlined in Table 1. The primary outcome measure is the Olerud & Molander Ankle Score, a functional outcome questionnaire [30]. The Olerud & Molander ankle score is a rating scale from 0 (totally impaired) to 100 (completely unimpaired) based on 9 items. The response to each of the 9 items is assigned a score and the summation of these scores makes up the overall score.

**Table 1:** Outcome measures by time-point for the AIM study

**Outcome**	**Baseline assessments**	**Theatre**	**6 weeks**	**6 months (Outcome assessor blinded)**
**Olerud & Molander ankle score**	+ (prior to injury status)		+	+
**EQ-5D**	+ (day before injury status and on the day of assessment)		+	+
**SF-12**	+ (prior to injury status)		+	+
**General health** - medical history, smoking, alcohol intake, allergies and medication	+			
**Social circumstances** - place of residence, mobility status and care requirements	+			
**Ankle range of movement** - goniometer measurement of dorsiflexion, plantarflexion, inversion and eversion, uninjured and injured ankles.			+	+
**Radiological measurements of fracture and ankle joint congruence**		+	+	+
**Theatre procedure data** - Time in and out of theatre, experience of operating surgeon, implants used, type of anaesthetic, complications		+		
**Patient satisfaction measure** - 2 questions (Likert-type scale)			+	+
**Health economics questionnaire**			+	+
**Timed ‘Get up and Go’ test**				+

+ indicates measure recorded.

Mobility, assessed using the Timed ‘Get up and Go’ test [33], has been included as an outcome at 6 months. The Timed ‘Get up and Go’ test is a test specifically designed for frail older people, it records time taken to get up from a chair, walk a short distance, turn, and sit down again. Performance tests are a recognised standard for measuring mobility and associations with important end points including risk of falling, functional decline and institutionalisation [34]. We will report and analyse the pain sub-scales of the Olerud & Molander Score and the EQ-5D separately. Health related quality of life will be assessed using the EQ-5D and SF-12. We will also note the date participants commence partial weight-bearing.

Cost-effectiveness will be measured by an economic analysis conducted alongside the study. The outcomes will incorporate the elements of duration of inpatient hospital stay, theatre time/implant costs, fracture clinic visits, additional treatment costs and social dependency/support change.

At six months a health professional, who is blind to treatment assignment, will complete the clinical measurements and ensure completion of the study questionnaires. We are confident that with safeguards it will be possible to blind the assessors to assignment. Presence or not of the surgical incision will be obscured by an opaque bandage applied by a research nurse/therapist prior to the participant meeting the blinded assessor. Patients unable to attend will be contacted by telephone, or visited at home. We will undertake an analysis of the success of the blinding strategy using outcomes from questions asking blinded assessors to indicate if they believe they know the intervention allocated and/or received.

### Radiological outcomes, complications and further surgery

For both groups radiographs will be taken post-operatively, at six weeks and six months. Fracture union and joint position will be assessed on standard anteroposterior (ankle mortise view) and lateral radiographs using standard measures of joint congruence, fracture angulation, fibular shortening and subluxation [35]. The radiographs will be reviewed centrally by a trained independent assessor. Measurements will then be verified by two independent surgeons. Any disagreement will be resolved by a radiologist.

Adverse events resulting from medical co-morbidities or anaesthesia (part of normal care) will only be recorded as adverse events and not reported as serious adverse events. Expected complications including wound breakdown, loss of fracture position, etc. will also be recorded as adverse events only. Other fractures sustained, further surgery or major illness resulting in disability in the study period will also be recorded. A serious adverse event will be any untoward medical occurrence that:

● Results in death within 30 days of surgery

● Results in death related directly to the surgical intervention at any time

● Life or limb threatening complication

● Re-hospitalisation (except a hospital stay for removal of syndesmosis screws which will be reported as an adverse event).

We plan to contact participants for extended follow up at least two years after intervention but this phase will be a separate sub-study.

### Randomisation

Randomisation will take place following screening and baseline assessments and the unit of randomisation will be the individual. We will use a remote 24-hour telephone randomisation service, computer generation of the allocation code once the participant is registered, ensures allocation concealment. Randomisation will be stratified by centre and fracture pattern, using infra/trans-syndesmotic (Weber A/B) and supra-syndesmotic categories (Weber C).

### Sample size

Given the paucity of data in the published literature the feasibility pilot data have been used as a primary source to inform estimates of variance and treatment effects measured using the Olerud & Molander score, and a range of secondary outcomes. We have tested the sensitivity of these estimates against data available in the published literature.

Although the original sample size estimate was based on a difference in proportions this was modified by the DMEC as data from the pilot study confirmed data to be normally distributed and that analysis based on a continuous score would be more efficient and meaningful. Parameters for the sample size were informed by data from the pilot study, known only to the study statisticians and DMEC. We utilised one-sided testing (p = 0.05) since we are not trying to prove that the new treatment is better than the standard, and gain considerable statistical efficiency [36]. Power was be set at 80% according to Food and Drug Administration (FDA) recommendations for bioequivalence studies [37].

Based on the mean difference observed between the groups of 2.6 points in the first 71 participants from the pilot study, pooled standard deviation of 16.2, an equivalence margin of +/- 6 points on the Olerud & Molander Ankle Score, yields a final sample size of 560 in total [36]. We inflated for loss to follow up of near 10% yielding a total sample size of 620. Published estimates to inform the selection of equivalence margins using the Olerud & Molander Score was non-existent. Using the pilot data to calculate standardised effects sizes, the equivalence margin includes small differences (<0.37) but excludes moderate or large treatment differences. This was consistent with clinical opinion supporting a 6 point margin excluding clinically important differences in this condition gathered in an informal survey of orthopaedic surgeons, and published data on the minimally clinically important differences for similar scores (Foot and Ankle Score, and visual analogue pain scores in acute injury) that report minimally clinically important differences greater than 10 points on a 100 point scale.

### Analysis of endpoints

In equivalence testing a maximum clinical difference (Δ_T_) is pre-specified at a level within which the two treatments can be considered not to differ in any clinically meaningful way. Therefore, the relevant null hypothesis is that a difference of greater than Δ_T_ exists in either direction, H0: Δ ≤ -Δ_T_ or Δ ≥ Δ_T_, and the trial is targeted at disproving this in favour of the alternative that no clinical difference exists, HA: -Δ_T_ < Δ < Δ_T_**.** FDA regulations recommend both a treatment received (per-protocol) and intention to treat analysis, aiming to demonstrate equivalence [37]. Use of an ITT approach as in a superiority trial sometimes increases the chance of falsely claiming equivalence [38,39]. Initially, a per-protocol analysis will be undertaken where only the patients who received their allocated treatment will be analysed and those patients who did not, will be excluded from the analysis. Following this an intention to treat analysis will be carried out where all randomised patients will be analysed according to the treatment they were randomised to.

The result of the analysis of the primary endpoint should be one of the following:

● The confidence interval for the difference between the two treatments lies entirely within the equivalence range, -Δ_T_ to Δ_T,_ so that equivalence may be concluded with only a small probability of error.

● The confidence interval covers at least some points that lie outside the equivalence range, so that differences of potential clinical importance remain a real possibility and equivalence cannot safely be concluded.

● The confidence interval is wholly outside the equivalence range (though this is likely to be rare).

As well as assessing if equivalence is demonstrated in either case this will also form part of an additional sensitivity analysis to assess the range of potential biases that could have resulted from loss to follow-up, protocol deviations, withdrawal (and mortality). Numerical and graphical summaries of all the data will be compiled, including descriptions of missing data at each level. Estimates of treatment effect will be reported with 95% confidence intervals and a figure showing confidence intervals and margins of equivalence will also be presented. Our main analytical methods will be generalised linear models, and all analyses will adjust for important baseline co-variants to maximise precision.

The Olerud & Molander Ankle Score at 6 months is the primary outcome in this study and will be compared between treatment groups as the dependent variable in a linear regression model for the primary analysis. The treatment difference will be based on the estimates of the adjusted means and 95% confidence intervals. The Olerud & Molander Ankle score will also be presented as an ordinal outcome in a secondary analysis using ordered logistic regression or non-proportional odds models, depending on the validity of the proportional odds assumption, will be carried out. Secondary outcome measures will be similarly analysed with logistic regression models being used for categorical data and linear regression models for continuous data. Time to event data (e.g. time to discharge) will be analysed using a log-rank test. Any patients who have not experienced an event at the time point of interest or withdrew will be censored. The proportion in each treatment group experiencing an event over time will be illustrated using a Kaplan-Meier curve. The p-value and a hazard ratio with its 95% CI from a Cox proportional hazards model will also be presented. The proportional hazards assumption across treatment arms will be checked graphically using a log-cumulative hazard plot. A data analysis plan will be agreed with the Independent Data Monitoring Committee.

### Economic analyses

The costs of the treatment will include implants, cast material, radiographs, surgical operating time, duration of hospital admission, rehabilitation, and post-operative care. Resource use will be collected during the follow up period and will consider major costs falling on the health service and personal social services (corresponding to the NICE reference case). We will also look at the broader societal perspective to include social services costs and costs falling on individual patients/carers. These will be valued in monetary terms by applying unit costs from standard sources such as the NHS Reference costs and the PSSRU Costs of Health and Social Care. The outcome measure will be the Quality Adjusted Life Year (QALY), based on the EQ-5D instrument with utility weights taken from the UK General Population tariff [40]. All costs and outcomes will be discounted at 3.5% per annum as per the NICE reference case [41].

Two timeframes will be considered for the economic evaluation - a six-month timeframe to correspond to the observed data from the clinical trial and a lifetime analysis which will be based on projection of the clinical trial data using decision-analytic modelling techniques [42].

Uncertainty for the six-month analysis corresponding to the period of the trial will be handled through non-parametric bootstrapping. Uncertainty for the additional parameters introduced as part of the modelling projection will be handled using probabilistic sensitivity analysis based on Monte Carlo simulation. Sensitivity of the analysis to individual parameter uncertainty as well as overall decision uncertainty will be assessed and presented [43].

A separate sensitivity analysis will explore the potential importance of including productivity (indirect) costs of patients/carers alongside direct costs in the societal perspective analysis. This analysis will be based on estimates of days lost from work in combination with alternative methods for valuing a day’s productivity.

### Qualitative study

In order to explore patient experience of their treatment and recovery, a purposive sample of 40 patients will be interviewed using a semi-structured interview schedule between six to ten weeks post-treatment. The sample will cover patients from: both treatment options; two study sites; a range of age, gender and accommodation. Participants will be fully informed and provide written consent. The interview will be conversational in style to allow patients to identify their experiences and the issues that concern them. The research question will be; what are the experiences of patients with an unstable ankle fracture? The key interview questions will cover what it is like to have an unstable ankle fracture; their experiences of treatment, what it is like living with a cast and their experience of treatment with surgery. This will be followed by prompts such as: tell me more about that; how did that affect you; how did you feel about that; how did you manage. To ascertain the impact of the trial on the participants they will also be asked, what is it like to take part in a trial. The interview will be taped and transcribed verbatim. Analysis will be line by line, identifying codes, building categories and themes, drawing on the work of Miles and Huberman [44]. NVivo7, a software package for qualitative data, will be used to help with data management. The intention is to understand how patients make sense of their treatment and recovery and whether there are any differences in experience between the two treatments.

### Ethics

The Oxfordshire Research Ethics Committee has given approval for this study. We will comply with the Medical Research Council Good Clinical Practice guidelines [45], and the trial will run under the Standard Operating Procedures of Warwick University Clinical Trials Unit and Oxford University. An independent data monitoring committee will meet twice yearly, with an option to increase if specific concerns arise.

### Reporting

The trial will be reported in accordance with the CONSORT statement [46] and its extensions relating to non-pharmologic [47], pragmatic [48] and equivalence trials [39].

## Discussion

This multicentre study was open to recruitment July 2010 and is due to be completed in December 2013.

## Abbreviations

AOUK: UK Association for Osteosynthesis; ORIF: Open Reduction and Internal Fixation; CCC: Close contact casting; NHS: National Health Service; QALY: Quality adjusted life year.

## Competing interests

The authors declare that they have no competing interests.

## Authors’ contributions

KW is the chief investigator, designed the study, contributed to writing the paper and is senior grant holder. DK is the research physiotherapist for the trial and contributed to the development of the study protocol and to writing the paper. LM is the trial manager and contributed to the development of the study protocol and reviewing the paper. BG designed the study and contributed to writing the paper. RH developed the clinical aspects of the trial. IP and TC developed the clinical aspects of the trial, reviewed the paper and are grant holders. ET designed the qualitative aspects of the trial, contributed to writing the paper and is a grant holder. RL and CK developed the statistical analysis plan for the study, contributed to writing the paper. AB designed the health economic aspects of the study, contributed to writing the paper and is a grant holder. SL designed the study, contributed to writing the paper and is a senior grant holder. All authors read and approved the manuscript.

## Pre-publication history

The pre-publication history for this paper can be accessed here:

http://www.biomedcentral.com/1471-2474/15/79/prepub

## Trial steering committee

Professor Margot Gosney *(Independent Chair),* Professor Keith Willett *(Chief Investigator)*, Professor Sallie Lamb *(Grant co-applicant)*, Professor Andrew Briggs *(Grant co-applicant and AIM Trial Health Economist)*, Professor Matt Costa *(Orthopaedic surgeon)*, Mr Chris Knox and Dr Ranjit Lall *(AIM Trial Statisticians)*, Dr Stephen Bremner *(Independent Member – Statistician)*, Ms Lesley Morgan *(AIM Trial Manager)*, Mr Tim Chesser *(Grant co-applicant)*, Professor Ian Pallister *(Grant co-applicant)*, Ms Rosamund Mengech (*Independent Member - Patient Representative).*
